# Perspective on Emerging Therapies to Achieve Functional Cure of Chronic Hepatitis B

**DOI:** 10.1007/s11901-024-00652-9

**Published:** 2024-02-10

**Authors:** Harish Gopalakrishna, Marc G. Ghany

**Affiliations:** 1 Liver Diseases Branch, National Institute of Diabetes and Digestive and Kidney Diseases, National Institutes of Health, 10 Center Drive, Building 10, Room 9B-16, Bethesda, MD 20892‐1800, USA

**Keywords:** Chronic hepatitis B, Novel treatments, Hepatitis B surface antigen loss, Functional cure, Antiviral therapy

## Abstract

**Purpose of Review:**

Advancements in our understanding of the hepatitis B viral (HBV) life cycle have paved the way for novel approaches to treat HBV infection. This review summarizes the various strategies being pursued to achieve a functional cure, defined as loss of hepatitis B surface antigen (HBsAg) and absence of viral replication 6 months off-therapy.

**Recent Findings:**

Direct acting antiviral, host targeting antiviral, and immunological approaches are in various stages of development as treatment for chronic HBV infection.

**Summary:**

Novel treatments are being developed in pursuit of a cure for HBV. Current evidence suggests a single therapeutic agent alone may be insufficient, necessitating the need for combination therapy targeting HBV and the host immune response. Ongoing research focused on identifying the best therapeutic combination holds promise in achieving functional cure for HBV.

## Introduction

Despite the availability of a safe and effective vaccine for over four decades, the global health burden of hepatitis B virus (HBV) infection remains high. Recent estimates suggest there are ~ 300 million people with chronic infection resulting in 820,000 deaths annually, primarily due to complications of cirrhosis and hepatocellular carcinoma (HCC) [1]. Additionally, there are approximately 1.5 million incident infections annually. Acknowledging this substantial health burden, the World Health Organization (WHO) has set an ambitious goal of reducing HBV incidence by 90% and HBV-related mortality by 65% by 2030 compared to 2015 levels [2].

Of the two approved classes of therapy for chronic hepatitis B (CHB), peginterferon alfa (Peg-IFN) and nucleos(t) ide analogs (NAs), NAs are the mainstay of treatment. NAs effectively suppress viral replication, improve hepatic inflammation, and reduce progression to cirrhosis and HCC [3]. However, only a small percentage of patients (≤ 1%) achieve the desired endpoint of functional cure, defined as the loss of hepatitis B surface antigen (HBsAg) and HBV DNA below the level of quantitation, that permits safe discontinuation of therapy [4, 5•]. Consequently, most patients require long-term therapy. This is because NAs do not directly target the two sources of HBsAg in serum-covalently closed circular DNA (cccDNA), a non-integrated form of the viral DNA located within the hepatocyte nucleus that serves as the template for viral transcription, and integrated HBV DNA [6, 7]. Although NAs have an excellent safety profile [8], long-term use is associated with increased healthcare costs, medication noncompliance, and risk of renal and bone toxicity. Therefore, there is a need for a novel, finite duration therapy that can achieve high rates of functional cure [9•].

This review focuses on novel therapies in development that hold promise for achieving functional cure. Advancements in therapeutics in combination with more widespread use of HBV vaccination will be crucial for reducing the global burden of HBV infection and achieving the WHO’s goal of HBV eradication.

## The HBV Viral Life Cycle

**Fig. 1:**
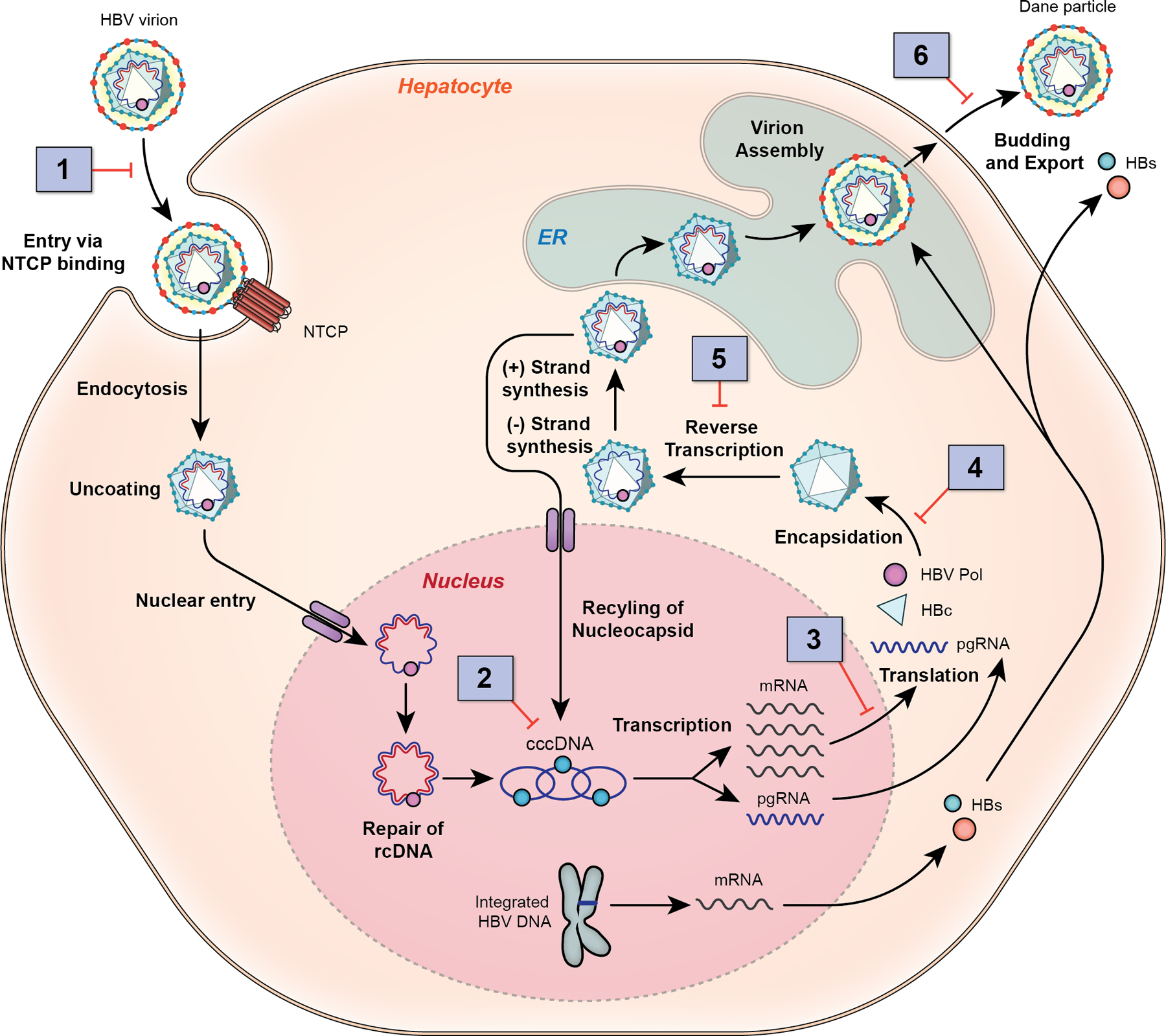
HBV life cycle and sites of drug targets. The viral life cycle begins with virion attachment to the hepatocyte surface via the sodium taurocholate co-transporting polypeptide (NTCP), which facilitates viral entry via endocytosis. After entry, there is uncoating and release of the partially double-stranded rcDNA, which is transported to the hepatocyte nucleus, where host cellular enzymes repair the rcDNA to form the cccDNA. cccDNA serves as the transcriptional template for all mRNAs, including the pregenomic RNA (pgRNA). In the cytoplasm, core proteins self-assemble and, through an encapsidation reaction, the pregenomic RNA and viral polymerase are packaged to form the nucleocapsid. Viral replication occurs within the nucleocapsid through reverse transcription to first form the negative strand then positive strand synthesis. The mature viral capsids containing rcDNA are then enveloped with the surface proteins in the endoplasmic reticulum (ER) and secreted from the infected cell as intact virions (Dane particle) or transported back to the nucleus to replenish the cccDNA pool. Drug targets (1) target viral entry, (2) cccDNA inhibition, (3) targeting viral translation, (4) capsid assembly modulators, (5) targeting the HBV polymerase (HBV Pol), and (6) targeting HBsAg secretion

HBV is a small hepatotropic DNA virus with a partially double-stranded circular genome of approximately 3.2 kilobases. The infectious virion or Dane particle consists of an outer lipid envelope made up of large, middle, and small HBsAg and an inner icosahedral viral nucleocapsid, consisting of core protein monomers with the viral DNA and HBV polymerase [10]. The viral life cycle and druggable targets are shown in Fig. 1.

### Treatment Goals and Endpoints

The ideal endpoint of treatment for HBV is a sterilizing cure. This is defined as the elimination of all traces of the virus including cccDNA and integrated HBV DNA [11]. However, achieving a sterilizing cure for HBV is currently not attainable. Therefore, the current focus of HBV treatment is aimed at obtaining a functional cure defined as sustained HBsAg loss for 24 weeks off-treatment, with or without seroconversion to anti-HBs, and HBV DNA below the limit of quantitation [12, 13]. A functional cure is associated with the reduction in clinical outcomes (cirrhosis, decompensated liver disease, and HCC), is durable off-treatment, and is easy to assess [14•, 15]. However, recent studies revealing that HBsAg can originate from both cccDNA and integrated HBV DNA highlight the complexity of achieving a functional cure [16, 17, 18••].

Given the challenges of achieving a functional cure, some have advocated for using a more achievable endpoint, partial cure, defined as HBsAg < 100 IU/mL, negative HBeAg status, and persistent HBV DNA levels below the lower limit of detection (LLOD) after 24 weeks off treatment [12]. Although a partial cure is also associated with the improvement in clinical outcomes, it is not a durable off-treatment endpoint. Additionally, it does not result in an HBsAg loss which is important for removing stigma for many patients. Thus, a partial cure is considered an intermediate endpoint, and a functional cure continues to be the preferred endpoint of novel therapies in the development for CHB [12].

### Novel Therapies in Development

As shown in Table 1, novel therapies in development for CHB can be categorized into two main groups: those that target key steps in the viral lifecycle, referred to as direct acting antiviral agents, and those that target the immune response, referred to as immunomodulators.

### Direct Antiviral Therapies

#### 1. Inhibitors of viral entry

Targeting the viral entry can block the infection of uninfected hepatocytes and thereby prevent the replenishment of the cccDNA pool. Bulevirtide, a small synthetic myristoylated lipopeptide mimicking the pre-S1 sequence of the large S protein, is an entry inhibitor which blocks HBV entry into hepatocytes by binding to the sodium taurocholate co-transporting polypeptide (NTCP) receptor [37]. Although there is evidence on the safety and efficacy of bulevirtide among subjects with chronic hepatitis D virus infection, there is only a limited data among patients with HBV mono-infection [19]. Bulevirtide has minimal effect on HBsAg levels, suggesting that its effectiveness in achieving a functional cure over a short dosing period may be limited. Hepalatide is another NTCP receptor blocker currently under evaluation [22].

Monoclonal and polyclonal antibodies that specifically bind to the N-terminal region of pre-S1 and block HBV attachment to hepatocytes are under development [38]. VIR-3434 is a monoclonal antibody designed to block the HBV entry into hepatocytes and reduce the level of virions and subviral particles. It may also function as a T cell vaccine against HBV. In a proof-of-principle study, VIR-3434 demonstrated a rapid HBsAg decline of ≥ 1 log IU/mL in virally suppressed subjects [21]. Studies in combination with pegIFN with or without a small interfering RNA (siRNA) and NA are ongoing [39]. Additionally, GC1102, a recombinant hepatitis B immunoglobulin, showed HBsAg loss in 2 (22%) out of 9 participants with low pretreatment HBsAg levels [22].

#### 2. Inhibitors of viral translation

Targeting HBV mRNA using siRNA or antisense oligonucleotides (ASO) are promising approaches to lower viral antigen production and indirectly inhibit HBV replication. siRNAs are short, double-stranded RNAs that lead to gene silencing in a sequence-specific manner by utilizing the endogenous RNA interference pathway. A single siRNA can target multiple HBV mRNAs due to shared terminal 3′ sequences in all HBV mRNAs. siRNAs require a carrier vehicle for their uptake as they do not readily cross the vascular endothelium. ASOs are single-stranded DNA or RNA sequences that bind to complementary sequences of mRNAs and degrade the mRNAs via a ribonuclease H–dependent mechanism. ASOs have the advantage of targeting pre-mRNA, enhancing their specificity and reducing off-target effects without the need for carrier vehicles [40].

### siRNAs

First-generation siRNAs, such as ARC-520, demonstrated the proof-of-principle reduction in HBsAg levels in chimpanzee and human studies. Notably, greater declines in HBsAg levels were observed in HBeAg-positive compared to HBeAg-negative chimpanzees. This was later shown to be as a result of changes in the target sequence of the mRNA encoded by integrated but not cccDNA-derived mRNA. In clinical trials, first-generation siRNAs (ARC-520 and ARC-521) showed modest reduction in HBsAg (~ 0.5 log IU/mL) in both HBeAg-positive and HBeAg-negative virally suppressed subjects [41]. However, the development of ARC-520/521 was halted due to hepatotoxicity attributed to the delivery vehicle.

Second-generation, GalNAc-conjugated siRNAs (JNJ-3989, VIR-2218, RG-6346, and AB-729) were developed to target mRNAs transcribed from cccDNA and integrated DNA and improve delivery. These agents have shown promising results with no significant safety concerns [20, 42, 43]. A 2–2.5 log_10_ IU/mL decline in HBsAg levels can be achieved after one to four doses, which persisted for months post-treatment. Almost all patients achieve at least a 1 log_10_ IU/mL reduction in HBsAg levels, which may be sustained up to 1 year after the last dose. However, the decline in the HBsAg level was observed to plateau around 20 weeks of treatment, raising concerns about the long-term efficacy of siRNA-based approaches. In a trial involving AB-729, the initial decline in HBsAg plateaued after a few weeks, and no participant achieved HBsAg loss. Mild ALT elevation and increased HBV-specific T cell activation markers were associated with HBsAg decline in some participants [23].

Given the limitations and absence of a functional cure with siRNA monotherapy, combination therapy is a logical next step. Combining AB-729 with NAs in HBeAg-negative participants demonstrated a marked and sustained decline in HBsAg and HBV DNA levels without ALT flares in participants who achieved HBsAg < 100 IU/mL [44]. In another study combining VIR-2218 with the monoclonal antibody VIR-3434, most participants achieved HBsAg level of < 10 IU/mL at the end of treatment, but none achieved a functional cure [45]. Another trial combining VIR-2218 with PegIFNα resulted in a greater decrease in HBsAg levels, compared to VIR-2218 monotherapy, 2.55 vs. 1.89 log_10_ IU/mL, with 15.6% of subjects achieving HBsAg loss by the end of treatment [24].

Many questions remain to be clarified with the use of siRNA in chronic HBV infection. The optimal duration is unknown, which agent to combine it with and in what sequence. Not all combinations will result in synergy as was observed in a trial combining a siRNA and capsid assembly modulator [46]. The results of several ongoing combination trials with various agents are eagerly awaited.

### ASOs

In preclinical studies, ASOs have shown promise in reducing serum HBsAg and HBV DNA levels [47]. In clinical trials, GalNAc-conjugated ASOs, RG6004 and GSK3389404, only resulted in moderate dose-dependent decline in HBsAg levels. These reductions were transient, and HBsAg levels rebounded to baseline within a few weeks after therapy cessation. Consequently, these ASOs are no longer in development [48, 49].

In a phase 2 trial of bepirovirsen, an unconjugated ASO, at a dose of 300 mg per week for 24 weeks, sustained HBsAg, and HBV DNA loss was observed in 9 to 10% of participants [25•]. ALT flares were noted in 40% of patients receiving bepirovirsen monotherapy. This was an important study demonstrating that a functional cure could be achieved with short-duration finite monotherapy. Similar to siRNAs, ASOs are safe and well tolerated.

Whether the sustained reduction in HBsAg levels achieved with siRNAs and ASOs will result in HBsAg clearance over time remains to be determined. Nevertheless, these are promising agents that likely will be part of a functional cure regimen.

#### 3. Inhibitors of capsid assembly

Capsid assembly modulators (CAMs), also known as core protein allosteric modulators (CpAMs), target the HBV core protein. By modifying nucleocapsid assembly, the site of genome replication, CAMs disrupt the viral life cycle and inhibit HBV replication. These compounds bind to a specific hydrophobic pocket between core protein dimers, inducing the formation of aberrant nucleocapsids or empty nucleocapsids, depending on the type of CAM [50]. Two types of CAMs have been described based on their mechanism of action: Type 1 CAMs (CAM-As) induce the formation of aberrantly assembled nucleocapsids, while type 2 CAMs (CAM-Es) result in the formation of morphologically normal but empty nucleocapsids [51].

CAMs offer several therapeutic advantages over other novel agents in development, including an oral route of administration and potent inhibition of viral replication across different genotypes. CAM-As, such as RO7049389, have shown significant reductions in HBV DNA and RNA levels in HBeAg-positive subjects [52]. CAM-Es, including vebicorvir, JNJ-6379, and JNJ-0440, have demonstrated similar declines in HBV DNA and RNA without significant effects on HBsAg levels [27, 53, 54]. CAMs alone do not have a measurable impact on HBV antigens and carry an increased risk of resistance, necessitating combination therapy with other antiviral agents. Similar to NAs, discontinuation of CAM treatment can lead to viral relapse and hepatitis flares.

Current CAMs must invariably be used as combination therapy due to concerns for resistance. When combined with NAs, CAMs demonstrate faster and greater inhibition of viral replication and transcription compared to NA alone. They can also achieve a deeper suppression of HBV DNA and HBV RNA in subjects already receiving NA treatment [55]. However, minimal changes were noted on HBeAg and HBsAg levels with vebicorvir alone or in combination with entecavir [55]. Unfortunately, vebicorvir is no longer in development because of recognized hepatotoxicity [56]. However, several other more potent second-generation CAMs including ABI-H2158, ABI-H3733, AB-836, ALG-000184 [57], and VNRX-9945 are currently under development and hold promise for greater efficacy and improved safety [58, 59].

CAMs have also been combined with siRNAs and NAs. The REEF1 trial evaluated a triple combination regimen of a siRNA (JNJ-3989), with a CAM (bersacapavir, formerly JNJ-6379), and NA. Bersacapavir alone demonstrated only minimal declines in HBsAg levels. Surprisingly, the triple combination regimen resulted in a lower reduction in HBsAg levels compared to the dual combination of siRNA and NA, after 48 weeks of treatment [60]. In the REEF-2 trial, non-cirrhotic HBeAg-negative HBV subjects on NAs received add-on treatment with bersacapavir and JNJ-3989 or placebo for 48 weeks. At the end of the treatment, the active treatment group showed a significant reduction in HBsAg levels, while the placebo group had minimal reduction. Although 71% of participants in the active treatment group achieved HBsAg levels below 100 IU/mL, none achieved HBsAg clearance. One subject experienced a severe hepatitis flare following withdrawal of NA therapy and required liver transplantation [61]. A promising third-generation CAM, AB-836 with potent inhibition of HBV replication (declines of 3.04 to 3.55 log_10_ IU/mL at day 28) depending on AB-836 dose, without significant HBsAg decline has been discontinued due to hepatotoxicity [59].

The lack of reduction of HBeAg and HBsAg with CAMs is a major limitation of this class of drugs. Thus, the exact role of CAMs in a curative regimen remains to be determined.

#### 4. Inhibitors of cccDNA

Permanent silencing or elimination of cccDNA is the sine qua non for achieving a cure for HBV infection and preventing the risk of reactivation [62]. Targeting cccDNA is a challenge due to its nuclear location. Nevertheless, there are several promising approaches in development. One is to inhibit the multiple steps in its formation from rcDNA, another is to prevent the nuclear import of rcDNA by capsid-targeting drugs. An alternate approach is to silence its transcriptional activity by inducing the host cell’s epigenetic machinery, or by blocking the de-silencing activity of HBV X protein (HBx). Finally, existing cccDNA may be degraded directly using genome editing with designer nucleases or indirectly through immune-mediated mechanisms, such as APOBEC enzymes [63].

Several candidate molecules have been shown in vitro to disrupt the conversion of rcDNA to cccDNA. CCC-0975, a disubstituted sulfonamide inhibitor, may inhibit cccDNA formation [64]. However, it primarily affects new cccDNA formation and does not impact the established pool of cccDNA. Another potential candidate, CCC_R08, is an oral cccDNA inhibitor that has shown sustained reductions in HBsAg and cccDNA in mouse models [65]. Further research is needed to explore the clinical effectiveness of such a strategy in the clinic.

Interferon-alpha and lymphotoxin-β-receptor activation have been shown to degrade cccDNA via APOBEC 3A and 3B-mediated deamination of the negative genomic strand. The Farnesoid X receptor (FXR-α) was shown to be a proviral host factor for HBV. Paradoxically, FXR-α agonists were shown to decrease cccDNA levels [66]. In early clinical studies, it was shown that the combination of vonafexor (EYP001), an FXR agonist, and Peg-IFN α may enhance the action of Peg-IFN α, based on the magnitude in reduction of HBsAg levels. This remains to be proven as there was no Peg-IFN α control arm [67].

cccDNA is organized into a chromatin-like structure, making it amenable to epigenetic manipulation. Gene editing methods, including zinc finger nucleases, transcription activator–like effector nucleases, and the CRISPR/Cas9 system, have been shown to degrade cccDNA and are promising strategies under investigation [68, 69]. However, challenges include the delivery of the editing tools to all infected hepatocytes, cleaving integrated HBV DNA, and the risk of off-target effects.

Targeting HBX could offer a durable approach to silencing cccDNA. Nitazoxanide, an antiparasitic agent, and pevonedistat, a neddylation inhibitor, have shown potential in inhibiting cccDNA formation by targeting HBX, a protein essential for viral transcription, and its interaction with DDB1 [70].

#### 5. Inhibitors of HBV polymerase

The HBV polymerase, the sole viral protein with enzymatic action, plays a critical role in viral replication. NAs widely used in HBV treatment primarily target the reverse transcriptase activity of the HBV polymerase [5•]. However, despite their effectiveness in inhibiting viral replication, they do not affect cccDNA and have a limited impact on HBsAg. Therefore, replication rapidly returns in a majority of patients once the NA is discontinued necessitating long-term treatment. Current efforts are focused on improving potency and reducing metabolite toxicity of newer NAs such as pradefovir [30, 31]. Targeting the other enzymatic function of the HBV polymerase, its RNAseH function, is being explored. RNAseH degrades the pgRNA after it serves as the template for negative strand synthesis. Although many RNAseH inhibitors have been identified, none is currently in clinical development.

#### 6. Inhibitors of HBsAg release

Clearance of HBsAg is the current goal of HBV therapies in development. Consequently, there is interest in developing agents that can reduce HBsAg levels. Nucleic acid polymers (NAPs) [33] are one potential approach being explored [71].

NAPs are synthetic oligonucleotides that selectively target the assembly and secretion of HBV subviral particles. They have little to no effect on mature virion secretion. By reducing circulating HBsAg levels, NAPs are believed to restore immunomodulation and promote host-mediated clearance [72]. In a small study, the addition of NAPs (REP 2139 or REP 2165) to a regimen of tenofovir and PEG-IFN resulted in sustained suppression of HBsAg to undetectable levels in 44% of participants. These participants also developed anti-HBs antibodies, suggesting either a potential restoration of the immune response or a shifting of the balance between levels of HBsAg and anti-HBs in circulation [33]. However, it is important to note that a majority of NAP-treated subjects experienced grade 3–4 ALT flares. Larger controlled studies are needed to further evaluate the efficacy and safety of NAPs.

A similar compound, S-antigen transport–inhibiting oligonucleotide polymers (STOPS), which is a locked nucleic acid-modified version of a NAP, is no longer in development due to the minimal effect on HBsAg levels [73].

### Immunomodulators

In chronic HBV infection, patients often have impaired innate and adaptive immune responses. High levels of viral antigens, such as HBsAg, can lead to the exhaustion of HBV-specific T cells, further compromising the immune response [74]. To achieve long-term control of HBV infection, it is crucial to restore immune function.

#### 1. Inducing innate immunity

In chronic HBV infection, the innate immune system has difficulty sensing and effectively responding to the HBV. Additionally, HBV may actively evade the innate immune response leading some to consider it a stealth virus [74]. A number of therapeutic approaches are being developed to restore the innate immune response. These include Toll-like receptor (TLRs) agonists, retinoid acid–inducible gene-I (RIG-I), and stimulator of interferon genes (STING), which aim to induce the production of interferons and proinflammatory cytokines that can help clear HBV [75].

TLRs play a critical role in the early innate immune response by sensing highly conserved bacterial or viral products such as LPS, bacterial DNA, and viral double-stranded RNA. The stimulation of TLRs results in a variety of cellular responses that lead to the production of IFNs, pro-inflammatory cytokines, and effector cytokines that eliminate pathogens. The TLR-7 agonists vesatolimod (GS-9620), RO7020531 and JNJ-4964, and TLR-8 agonist selgantolimod (GS-9688) [34, 76] have shown promise in preclinical trials by activating intrahepatic dendritic cells and triggering the production of interferons, as well as activate natural killer (NK) cells and T cells [77, 78]. However, clinical trials with vesatolimod and selgantolimod did not show substantial benefit in reducing HBsAg levels [34, 76]. In a phase 2 study, selgantolimod led to HBsAg loss in 5% of virally suppressed subjects and a modest reduction in HBsAg levels [34].

Inarigivir (SB9200), an oral agonist of RIG-I and NOD2 that induces interferon-mediated antiviral immune responses in virus-infected cells, is no longer being developed due to hepatotoxicity and death of a research subject [79], 80.

Further research is needed to define the optimal innate immune targets, and to establish the role of agonists of innate immunity in combination with antiviral therapy. Moreover, understanding the timing and duration of immune modulation in relation to viral inhibition will be important for achieving a functional cure.

#### 2. Inducing adaptive immunity

Restoring an adequate adaptive immune response will likely be necessary for controlling and potentially curing HBV infection.

Checkpoint inhibition, using antibodies targeting the programmed death receptor (PD-1) or programmed death ligand (PD-L1), is one non-specific approach to restore T cell function. PD-1 and its ligands downregulate T cell activation. PD-1 has been shown to be overexpressed by HBV-specific T cells. Thus, agents that block PD-1 and its ligand checkpoint inhibitors represent a therapeutic strategy to restore function of HBV-specific T cells and enhancing antibody production [81]. Proof-of-concept has been demonstrated in cancer treatment. There is, however, a concern for autoimmunity, and an uncontrolled immune response may lead to flares of hepatitis. In a pilot study, the use of PD-1 inhibitor nivolumab with or without GS-4774, a therapeutic vaccine in virally suppressed subjects, resulted in only modest declines in HBsAg levels. HBsAg seroclearance was achieved in one patient in the nivolumab-only group [82]. In another phase 2 study using the PD-L1 antibody envafolimab (ASC22), 3 out of 7 subjects (43%) with baseline HBsAg levels below 100 IU/mL achieved HBsAg loss [35]. Oral, liver-specific checkpoint inhibitors are in development. The optimal duration and sequence of checkpoint inhibition in a therapeutic regimen remain to be determined.

Strategies to restore T cell responsiveness include creation of genetically engineered T cells. T cell receptors cloned from HBV-reactive cells can be introduced into T cells. Alternatively, T cells can be modified to express chimeric antigen receptors that specifically target HBV antigens on the hepatocyte surface [83]. These genetically modified T cells have been shown to effectively eliminate HBV-infected cells in vitro and have shown safety in animal models [84, 85]. The safety and efficacy of using genetically engineered T cells were shown in a small study in which adoptive transfer of autologous T cells expressing HBV-specific T cell receptors was achieved in participants with HBV-related HCC [86]. This may open the possibility of using this approach in patients with CHB.

#### 3. Therapeutic vaccines

Therapeutic vaccination aims to induce both B and T cell immune responses, as the resolution of acute HBV infection is associated with lifelong immunity mediated by these components of the immune system. Previous attempts to develop a therapeutic vaccine using a variety of antigens, protein-based, protein-antibody complex–based, and DNA-based vaccines, have been unsuccessful [87]. Novel vaccination strategies are exploring use of viral recombinant vectors, dendritic cell vaccines, and mRNA vaccines. Viral vector-based vaccines, in particular, offer the advantage of intracellular antigen expression, triggering a robust cytotoxic T lymphocyte response [88].

TG1050, an adenovirus 5–based vaccine expressing HBV antigens, and lentiviral vectors encoding HBV antigens, have demonstrated the induction of specific T cell responses but only modest declines in HBsAg levels [89]. Studies involving GS-4774, a yeast-based vaccine expressing HBsAg, HBcAg, and HBX, have shown minimal reduction in serum HBsAg levels, despite robust cytokine production by HBV-specific CD8 T cells [90].

Innovative strategies are being developed to enhance the immunogenicity of peptide-based vaccines. These include the utilization of novel nasal formulations such as NASVAC and derivatives of Sci-B-Vac, like BRII-179 [36, 91]. Clinical trials evaluating the safety and efficacy of these approaches have yielded promising results. The mucoadhesive carboxy-vinyl polymer (CVP-NASVAC) nasal therapeutic vaccine, containing HBsAg and HBcAg, has demonstrated significant reductions in HBsAg levels in participants receiving both NA therapy and HBV carriers. Notably, 9.5% of subjects (6 out of 71) achieved a functional cure [91]. Another study investigating BRII-179, which encompasses all three HBV surface envelope proteins, has shown moderate antibody responses; however, it did not lead to a noticeable reduction in HBsAg levels in virally suppressed subjects under NA therapy [36].

Prime-boost vaccination, involving sequential administration of vaccines with different antigens and delivery systems, is one of the more promising approaches to overcome HBV-specific tolerance and have been shown to generate high levels of memory T cells in preclinical studies. Additionally, combining viral antigenemia knockdown using siRNAs with therapeutic vaccination has resulted in the development of polyfunctional HBV-specific CD8 +T cells and elimination of HBV [92].

An advantage of vaccination is its theoretical safety, as it specifically stimulates HBV-specific immunity without non-specific activation of innate responses or autoimmunity. However, important considerations include determining the optimal vaccine composition, use of T or B cell adjuvants, the vaccine regimen, and the mode of administration need to be addressed in future studies.

## Combination Therapy

**Fig. 2:**
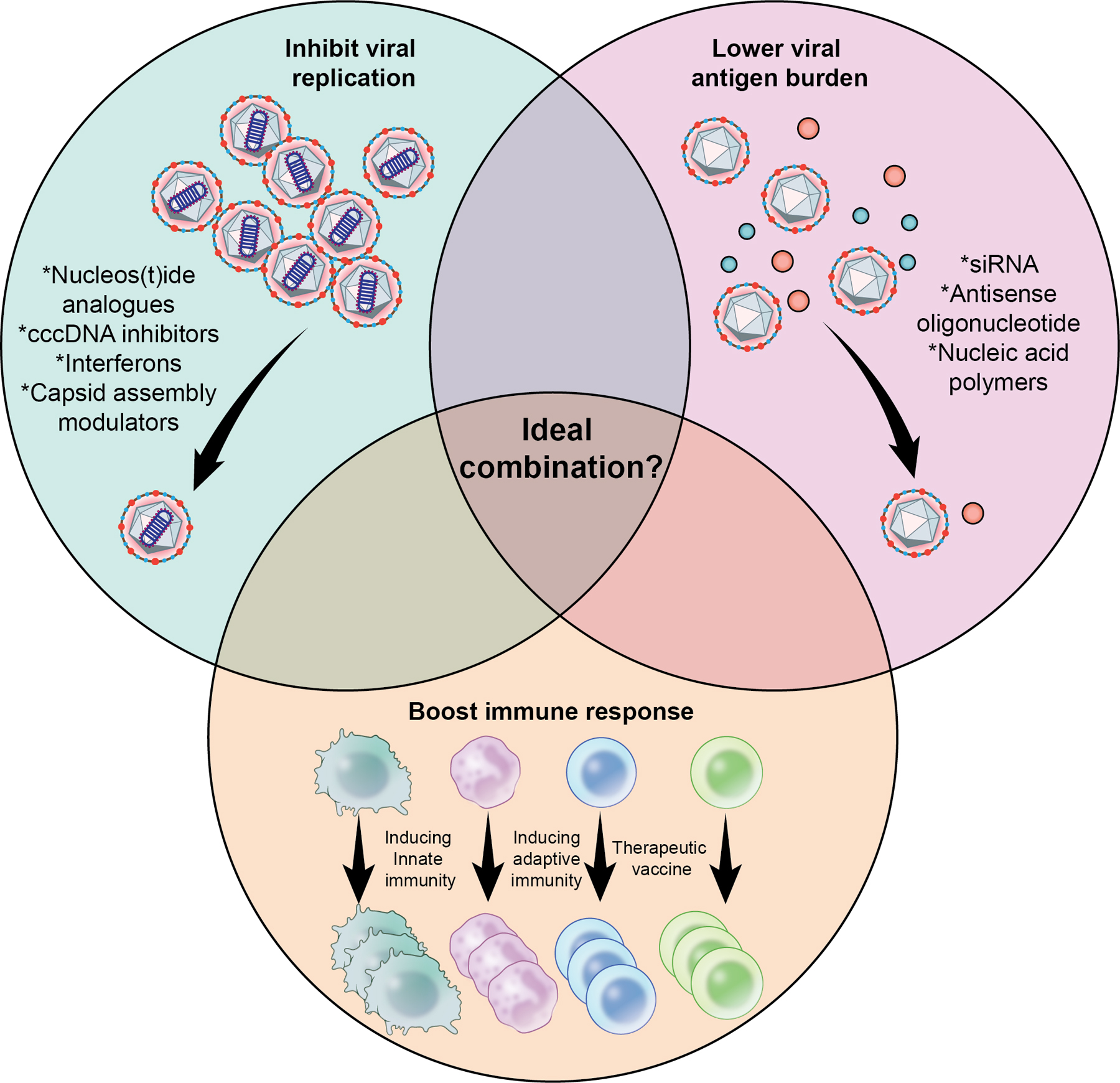
Possible therapeutic combinations for achieving a functional cure. Ideal combination is yet to be determined

Success with combination therapy for other infectious diseases such as those caused by human immunodeficiency virus and hepatitis C virus implies that a similar approach will be needed to achieve a functional cure of chronic HBV infection. Although over 40 compounds with multiple mechanisms of action are currently in development, Table 1, combination approaches are largely empirical due to the lack of knowledge of the mechanisms required to achieve viral control and HBsAg loss. The most promising approaches focus on agents that inhibit viral replication, reduce antigenic burden, and partially enhance/restore immune control against HBV, Fig. 2 [24, 25•, 33, 35, 45]. However, determining the optimal combinations, timing and sequence of use, and treatment duration remains a challenge. Personalized treatment approaches may be necessary based on factors such as HBeAg status, viral load, HBsAg levels, and presence of cirrhosis.

## Conclusion

Combination therapy with currently approved agents peg-IFN and NAs can achieve functional cure in ~ 10% of patients. However, many patients decline or are ineligible to receive peg-IFN due to its poor tolerability. Functional cure rates with NAs alone range from 1 to 3%. This highlights the need for newer therapy that can achieve higher rates of a functional cure to reduce the morbidity and mortality associated with chronic HBV infection. Given that the bulk of patients with chronic HBV infection reside in low- and middle-income countries, the ideal regimen would be one that is of finite duration, orally administered, easy to manufacture, and scalable. To realize this goal, continued research, investment, and commitment from both the industry and the scientific community will be crucial.

## Figures and Tables

**Table 1:** Novel therapies for hepatitis B treatment

Drug target*	Agent	Route of administration and dose range	Phase of development
Direct acting antiviral agents			
Viral Entry inhibition			
Block NTCP receptor	Bulevirtide	SQ, 2–10 mg daily	Phase III [19]
	Hepalatide	SQ, 2.1–6.3 mg weekly	Phase II [20]
Monoclonal antibody blocking pre-S1 domain	VIR-3434	SQ, 6–75 mg	Phase I/II [21]
Viral translation			
Small interfering RNA	RG-6346	SQ	Phase II [22]
	JNJ-3989	SQ, weekly	Phase II [20]
	AB-729	SQ, 60 mg every 8 weeks	Phase II [23]
	VIR-2218	SQ, 20–200 mg 4 weeks apart	Phase II [24]
	ALG-125755	SQ	Phase I [22]
Antisense oligonucleotide	Bepirovirsen	SQ, 300 mg weekly	Phase III [25•]
Capsid assembly			
Capsid assembly modulators	ABI-H3733	Oral, 25 or 100 mg	Phase I [22]
	ABI-4334	Oral, 200 mg daily	Phase I [22]
	GLS4	Oral, 120 mg three times	Phase II [26]
	Bersacapavir	daily	Phase II [27]
	EDP-514	Oral, 25–250 mg daily	Phase I [22]
	RG7907	Oral, 200–800 mg daily	Phase II [28]
	Canocapavir	Oral, 50–200 mg daily	Phase II [29]
	ALG-000184	Oral, 100–300 mg daily	Phase I [22]
Covalently closed circular DNA inhibitor			
Farnesoid X receptor agonist	ASC42	Oral, 10–15 mg daily	Phase II [22]
HBV polymerase			
Prodrugs of NA	Pradefovir	Oral, 30–75 mg daily	Phase II [30]
	HS-10234	Oral, 25 mg daily	Phase III [31]
Nonchain terminating NA	ATI-2173	Oral, 10–50 mg daily	Phase II [32]
HBsAg secretion inhibitors			
Nucleic acid polymers	REP-2139	IV, 250 mg weekly	Phase I/II [33]
Immunomodulators			
Inducing innate immunity			
Toll like receptor 7 agonists	RO7020531	Oral, 150 mg daily	Phase II [22]
Toll like receptor 8 agonists	Selgantolimod	Oral 1.5–3 mg weekly	Phase II [34]
Inducing adaptive immunity			
Soluble bispecific T cell receptor	IMC-I109V		Phase I [22]
Checkpoint inhibitor	ASC22	SQ 0.3–2.5 mg/kg	Phase II [35]
Therapeutic vaccine	BRII 179 (VBI-2601)	IM	Phase II [36]
	HepTcell	IM	Phase II [20]
	GSK3528869A	IM	Phase I/II [20]
	VVX001	SQ	Phase II [20]
	VTP-300	IM	Phase II [20]

* Only therapies currently in clinical trials are included

*NTCP* sodium taurocholate co-transporting polypeptide, *SQ* subcutaneous, *mg* milligram, *RNA* ribonucleic acid, *DNA* deoxyribonucleic acid, *NA* nucleos(t)ide analogs, *IV* intravenous, *IM* intramuscular

## Abbreviations

AASLD: American Association for the Study of Liver Disease
ALT: Alanine aminotransferase
ASO: Antisense oligonucleotides
AST: Aspartate aminotransferase
CAMs: Capsid assembly modulators
CHB: Chronic hepatitis B
cccDNA: Covalently closed circular DNA
DNA: Deoxyribonucleic acid
dslDNA: Double-stranded linear DNA
EASL: European Association for the Study of the Liver
FXR-α: Farnesoid X receptor
HBcrAg: Hepatitis B core antigen
HBeAg: Hepatitis B e antigen
HBsAg: Hepatitis B surface antigen
HBV: Hepatitis B virus
HBx: Hepatitis B virus X protein
HCC: Hepatocellular carcinoma
HSPG: Heparan sulfate proteoglycans
IU: International units
IVDU: Intravenous drug users
kb: Kilobase pairs
LLOD: Lower limit of detection
mL: Milliliters
NAs: Nucleos(t) ide analogs
NTCP: Sodium taurocholate co-transporting polypeptide
NAPs: Nucleic acid polymers
pgRNA: Pre-genomic RNA
PEG-IFN α: Pegylated interferon alfa
PCR: Polymerase chain reaction
RIG-I: Retinoid acid inducible gene-I
RNA: Ribonucleic acid
rcDNA: Relaxed circular DNA
STING: Stimulator of interferon genes
STOPS: S-Antigen transport-inhibiting oligonucleotide polymers
siRNA: Small interfering RNA
TLR: Toll-like receptor
WHO: World Health Organization
